# ZnO and SiO_2_ nanopriming enhancing seedling performance and changing volatile profiles of *Melissa officinalis* under drought and salinity stress

**DOI:** 10.3389/fpls.2026.1980257

**Published:** 2026-10-09

**Authors:** Sam Mokhtarzadeh

**Affiliations:** Department of Crop and Animal Production, Vocational School of Food, Agriculture and Livestock, Bingol University, Bingol, Türkiye

**Keywords:** drought, lemon balm, nanopriming, salinity, volatiles

## Abstract

**Introduction:**

*Melissa officinalis* L. is an important medicinal and aromatic plant whose germination, seedling establishment, and secondary metabolism can be adversely affected by drought and salinity. This study investigated whether ZnO and SiO_2_ nanopriming could mitigate these effects and modify the volatile profile of *M. officinalis*.

**Methods:**

Two independent factorial experiments were conducted under drought and salinity stress. Seeds were hydroprimed as the control or nanoprimed with ZnO or SiO_2_ at 50 or 100 mg L^−1^. Drought treatments were 0, −0.2, −0.4, and −0.6 MPa, while salinity treatments were 0, 4, 8, and 12 dS m^−1^. Germination and early seedling traits were evaluated. Selected 50 mg L^−1^ treatments under moderate stress conditions were subsequently subjected to volatile profiling by GC/GC-MS.

**Results:**

Nanopriming significantly improved several germination and early seedling traits, with the strongest responses generally observed at 50 mg L^−1^. ZnO at 50 mg L^−1^ increased germination from 42% in the non-primed control to 56% under −0.4 MPa drought and from 36% to 52% under 8 dS m^−1^ salinity. Low-dose ZnO and SiO_2_ also improved radicle and plumule growth and seedling vigour, although their effects declined under severe stress. Eleven volatile compounds were detected in all analyzed samples, with geranial and neral as the predominant constituents. Alloaromadendrene showed the largest descriptive change, increasing from 2.4% in the control to 6.3 -6.8% in selected treatments. Because volatile analysis was performed once per selected treatment (n = 1), these compositional differences were interpreted descriptively.

**Discussion:**

Low-dose nanopriming, particularly ZnO at 50 mg L^−1^, improved early-stage tolerance of *M. officinalis* to independently imposed drought and salinity stress and was associated with changes in its volatile profile. Replicated chemical analyses and greenhouse and field experiments are required to confirm the observed volatile responses and agronomic applicability.

## 1 Introduction

*Melissa officinalis* (lemon balm) is one of the most important medicinal and aromatic plants of the Lamiaceae family, which is widely used in the pharmaceutical, cosmetic, and food industries due to its valuable compounds such as citral, geraniol, citronellal, and rosmarinic acid (Mokhtarzadeh et al., 2017). The preservation of plant productivity, yield, and chemical purity of essential oils in the face of environmental stress is thus critical for the consistent production of *M. officinalis* as a medicinal and aromatic crop. Environmental stresses, especially drought and salinity, are among the most important factors limiting growth and production of secondary metabolites in medicinal plants. In recent years, nanotechnology in agriculture has been proposed as an effective tool for increasing plant tolerance to environmental stresses (Imtiaz et al., 2023; Mridha et al., 2025). Nanopriming, as one of the new methods for improving germination and increasing plant tolerance to environmental stresses, can have a significant impact on the quantity and quality of essential oils of medicinal plants. Drought and salinity have been shown in M. officinalis to limit growth and biomass accumulation while also negatively impacting photosynthetic performance and mineral nutritional status (Razavizadeh et al., 2023). However, these stresses can sometimes cause a relative increase in some essential oil compounds by stimulating defense pathways and increasing secondary metabolites (Farahani et al., 2025). Nanopriming of seeds with nanoparticles such as nano-zinc oxide (ZnO), nano-iron oxide (Fe_2_O_3_), nano-silicon dioxide (SiO_2_), and nano-titanium oxide (TiO_2_) improves germination, increases antioxidant enzyme activity, and reduces oxidative stress-induced damage. This process increases volatile biosynthesis capacity by increasing water availability, improving cell membrane permeability, and regulating the expression of genes related to secondary metabolite synthesis (Jadczak et al., 2020; Abbasi Khalaki et al., 2021; Imtiaz et al., 2023). ZnO and SiO_2_ were chosen for comparison because they are functionally distinct nanopriming agents that can influence plant stress tolerance. ZnO can offer Zn, an essential micronutrient involved in enzyme function, membrane integrity, and antioxidant defence. SiO_2_ has been linked to enhanced water interactions, structural stability, and protection against oxidative damage during abiotic stress. Individual comparisons of these nanoparticles provide a chance to discover whether their diverse mechanisms of action cause different seedling responses to drought and salinity. Under drought stress conditions, nanopriming usually increases the total volatile composition compared to stressed control plants. This reaction could be linked to previously identified mechanisms, such as photosynthesis maintenance, enhanced proline accumulation, improved plant hydration status, and terpenoid biosynthesis modulation. However, the mechanisms involved were not directly assessed in this investigation (Ochoa-Chaparro et al., 2025). Also, under salinity conditions, nanopriming prevents severe yield reduction and maintains volatile profile at a higher level by reducing the accumulation of toxic sodium and chloride ions and increasing potassium and calcium absorption (Salve et al., 2025). Previous research has shown that nanopriming can affect secondary metabolism and volatile compound profiles in medicinal and aromatic plants; however, the extent and direction of these responses vary depending on the plant species, nanoparticle type and concentration, and stress conditions. In addition to increasing essential oil content, positive changes in the chemical composition of the essential oil have also been observed (Abbasi Khalaki et al., 2021). The nanopriming responses were examined under increasing stress intensities, including drought at osmotic potentials of 0, -0.2, -0.4, and -0.6 MPa, and salinity at EC levels of 0, 4, 8, and 12 dS m^-1^. These stress levels were chosen to compare the responses of non-stressed seedlings to increasing drought and salt stress. However, the comparative effects of nanopriming with ZnO and SiO_2_ on the seedling performance and volatile chemical profiles of *M. officinalis* under drought and salinity are still little understood. It is unclear if these two nanoparticles differ in their potential to boost early seedling responsiveness and affect volatile chemical profiles under different drought and salinity stress levels. Therefore, nanopriming can be used as an effective strategy to reduce the negative effects of drought and salinity stresses and improve essential oil yield in *M. officinalis* seedlings. This technology increases stress tolerance, preserves plant growth and promotes the synthesis of secondary metabolites, increasing quantity and improving quality of essential oil. This research hypothesized that the effects of ZnO and SiO_2_ nanopriming on germination and early seedling performance would vary according to the intensity of stress, with nanopriming counteracting the negative impacts of rising drought and salinity stress. The goal of this study was to compare the effects of ZnO and SiO_2_ nanopriming at 50 and 100 mg L^-1^ on germination percentage, radicle length, plumule length, and seedling vigour index of *M. officinalis* under independently imposed drought and salinity stresses, as well as to characterise the volatile compound profiles of selected low-dose nanopriming stress treatments.

## 2 Materials and methods

### 2.1 Plant material

The seeds of *M. officinalis* L. were obtained from Anoxymer. Ltd., Germany, 2024 and stored in a cold, dry place until needed for germination as per the established protocols for germination of balm. For aseptic germination, seeds were surface sterilised using 5% sodium hypochlorite for five minutes and rinsed three times with distilled water for five minutes each to eliminate any remaining sodium hypochlorite (Mokhtarzade et al., 2017).

### 2.2 Nanoparticles and priming treatments

Zinc oxide (ZnO) and silicon dioxide (SiO_2_) nanoparticles were commercially obtained from Merck (Sigma-Aldrich/Merck KGaA, Darmstadt, Germany; catalog nos. 677450 and 637238, respectively). According to the manufacturer’s specifications, ZnO was supplied as a nanopowder with a particle size of <50 nm (BET) and a purity of >97%, whereas SiO_2_ was supplied as a nanopowder with a particle size of 10–20 nm (BET) and a purity of 99.5% on a trace-metals basis. Stock suspensions were formulated in distilled water with 0.1% Tween-20 to improve dispersion stability, adhering to recognized NP-priming methodologies. In this research nanopriming treatments consisted of five groups that were control (hydropriming with distilled water containing 0.1% Tween-20), ZnO at 50 mg L^-1^, ZnO at 100 mg L^-1^, SiO_2_ at 50 mg L^-1^ and SiO_2_ at 100 mg L^-1^. The four nanoparticle treatments (ZnO at 50 and 100 mg L^-1^ and SiO_2_ at 50 and 100 mg L^-1^) and the hydroprimed control were evaluated at different drought and salinity levels. To compare the concentration-dependent beneficial or inhibitory effects, two different levels of nanoparticle exposure were chosen, 50 and 100 mg L^-1^, in the ranges previously used in *M. officinalis* nanoparticle studies and seed-nanopriming studies of other plant species with ZnO (Babajani et al., 2019; Razavizadeh et al., 2023). The 100 mg L^-1^ treatments were used in the main factorial studies, not as a preliminary screening step. The seeds were added to 100 mL conical flasks containing the appropriate priming solution at a seed-to-solution ratio of 1:5 (w/v) and incubated for 8 hours with continuous shaking at 100 rpm (Khan et al., 2022). The primed seeds were air dried in shade for 24 hours under laboratory conditions (25 ± 2 °C and 50-60% relative humidity) (Ochoa-Chaparro et al., 2025; Donia and Carbone, 2023).

### 2.3 Drought stress treatments

Utilizing the equation derived by Michel and Kaufmann (1973), a standard method in laboratory environments, drought stress was induced using PEG-6000 solutions corresponding to osmotic potentials of 0, -0.2, -0.4, and -0.6 MPa. (Okçu et al., 2005). In accordance with established drought simulation techniques, preconditioned seeds were placed in sterile Petri dishes containing two Whatman No. 1 filter papers, each imbued with 5 mL of PEG solution (Ochoa-Chaparro et al., 2025).

### 2.4 Salinity stress treatments

Salinity stress was induced using sodium chloride (NaCl) solutions of electrical conductivity (EC) levels of 0, 4, 8 and 12 dS m^-1^. The EC measurements were verified by an EC meter calibrated at 25 ± 1 °C. This method is consistent with the well-established protocols for evaluating salinity stress in germination studies (Szekely-Varga et al., 2020; Okey et al., 2021). The experimental design was similar to the drought assay. Five milliliters of each saline solution were used to saturate filter sheets in Petri dishes with seeds.

### 2.5 Germination conditions

In a growth chamber with a photoperiod of 12 hours of light and 12 hours of darkness, germination experiments were carried out at 25 ± 1 °C (Georgiu et al., 2025). To stop evaporation, Petri dishes were covered with parafilm. For 21 days, germination was tracked every day and solutions were added as needed. When the radicle was 2 mm or longer, germination was noted (Ochoa-Chaparro et al., 2025).

### 2.6 Experimental design

Drought and salinity studies were conducted separately in completely randomised factorial designs (CRD). For each combination of NP x stress treatment, three independent replicate Petri dishes (biological replicates) with 25 seeds per plate were used, for a total of 75 seeds per treatment combination. The experimental unit for the statistical analysis was the Petri dish, not the individual seed (n=3). Each experiment comprised five levels of nanopriming (NP) treatment and four levels of respective stress factor. Thus, two factorial experiments were carried out: nanoparticle priming × drought stress (5×4) and nanoparticle priming × salinity stress (5×4); drought and salinity were not imposed simultaneously. In each experiment, replicate Petri dishes for each treatment combination were randomly put in the growth chamber.

### 2.7 Measured germination and seedling parameters

Using established formulas from seed biology and priming research, the following germination and seedling traits were calculated:

• Germination percentage (GP, %):

*GP = (Number of germinated seeds at 21 days/Total number of seeds) × 100* (Ochoa-Chaparro et al., 2025).

• Seedling length (cm):

At day 21, radicle length (RL) and plumule length (PL) were measured in accordance with widely accepted measurements of seedling vigor (Georgiu et al., 2025).

• Seedling vigor index (SVI):

Seedling vigor index (SVI) was calculated as *SVI=GP×(RL+ PL)*, where GP is germination percentage, RL is mean radicle length, and PL is mean plumule length (Abdul-Baki and Anderson, 1973).

### 2.8 Statistical analysis

The data for the drought and salinity studies were analysed independently using Factorial ANOVA. The drought experiment examined the impact of nanoparticle priming (NP; 5 levels), drought stress (D; 4 levels), and their interaction (NP × D). The salinity experiment assessed the primary effects of nanoparticle priming (NP; 5 levels), salinity stress (S; 4 levels), and their interaction (NP × S). The experimental unit was a Petri dish, with three independent replicates for each treatment combination (n = 3). If significant effects were observed, means were compared using Duncan’s Multiple Range Test (p < 0.05). SAS (version 9.4) was used to conduct statistical analysis.

### 2.9 Microdistillation, GC/GC/MS analysis, isolation of the volatiles and their diagnosis

The seedlings were observed for 21 days before the samples for microdistillation were collected. The composition of the control sample and the sample under moderate drought and salinity stress treated with ZnO at 50 mg L^-1^ and SiO_2_ at 50 mg L^-1^ was determined based on the experimental data. The highest stress levels (-0.6 MPa drought and 12 dS m^-1^ salinity) were included in the germination and seedling-performance analyses but were not selected for volatile profiling. In this research 500–750 mg of dried balm herbage were microdistilled using the method outlined by Mokhtarzadeh et al. (2017). In order to collect volatile components, the process accumulation vial contained 350 μl of n-hexane that had been cooled to -5 °C and 2.5 mg of NaCl that had been dissolved in 0.5 ml of water. Following that, GC and GC/MS analysis was carried out as detailed by Mokhtarzadeh et al. (2017) in order to separate and degrade each organic layer in the accumulation vial concurrently. To keep the injector temperature at 250 °C, the split ratio was changed to 40:1. At 70 eV, mass spectra were captured. The mass range variation was set at m/z 35-450. The Agilent 6890N GC system with a 300 °C FID detector was used to perform the gas chromatography separation. A replica of a comparable segment was subjected to synchronous auto-injection for the same order of elution by GC/MS under identical working conditions. percentage of the separated chemicals’ relative quantities in FID chromatograms Correlating relative retention times with known samples or cross-referencing the relative index (RRI) to a series of n-alkanes with computer coordination against commercial (Wiley GC/MS Library, MassFinder 3 Library) (Koenig et al., 2004; McLafferty and Stauffer, 1989) was used to identify volatile components. The diagnosis was also determined by MS-known published literature (Joulian and Koenig, 1998; ESO, 2000). For the volatile-profile analysis, one GC/GC-MS determination was performed for each selected treatment condition (n=1). Therefore, the relative abundances obtained from FID peak areas were used for descriptive comparison only, and no inferential statistical analysis was performed on the volatile-composition data. Accordingly, SD and SE values could not be calculated for these measurements.

## 3 Results

The factorial ANOVA results (**Tables 1**, **2**) indicates that nanopriming (NP), drought stress (D), and salt stress (S) applied very significant impacts (p<0.05 or p<0.01) on all germination and seedling parameters, including germination percentage (GP), root length (RL), Plant length (PL), and seedling vigor index (SVI). Increasing levels of drought and salinity stress caused progressive declines in all measured parameters. The 50 mg L^-1^ ZnO and SiO_2_ treatments showed significantly higher overall means than the non-primed control for all assessed traits, whereas the 100 mg L^-1^ treatments showed intermediate responses and did not differ significantly from either the 50 mg L^-1^ treatments or the control (**Table 3**). The germination characteristics were consistently better at 50 mg L^-1^ (ZnO at 50 and SiO_2_ at 50) than at 100 mg L^-1^. High NP concentrations (100 mg L^-1^), on the other hand, shown restricted or inhibitory effects, particularly in situations of extreme stress.

**Table 1:** ANOVA (F- value) of *M. officinalis* seed germination traits affected by nanoparticle priming and drought.

Source of variation	df	Germination %	Plumule length	Radicle length	SVI
Nanoparticles (NP)	4	5.41**	4.96**	5.12**	6.38**
Drought (D)	3	208.97**	296.10**	265.46**	274.70**
NP × D	12	3.78*	3.66*	2.88*	4.19**
Error	40	—	—	—	—
CV (%)	—	11.7	16.3	19.2	19.6

*Significant at p<0.05 level.

**Significant at p<0.01 level.

**Table 2:** ANOVA (F- value) of *M. officinalis* seed germination traits affected by nanoparticle priming and salinity.

Source of variation	df	Germination %	Plumule length	Radicle length	SVI
Nanoparticles (NP)	4	4.67**	3.93**	4.31**	5.54**
Salinity (S)	3	176.55**	241.77**	221.31**	241.55**
NP × S	12	3.21*	3.12*	2.54*	3.88**
Error	40	—	—	—	—
CV (%)	—	12.8	18.9	19.3	21.4

*Significant at p<0.05 level.

**Significant at p<0.01 level.

**Table 3:** Nanoparticle mean values.

Nanoparticle	Germination (%)	Plumule	Radicle	SVI
Control	52.4b	1.82b	2.01b	96.4b
ZnO 50	62.1a	2.41a	2.55a	124.3a
ZnO 100	57.5ab	2.15ab	2.38ab	112.8ab
SiO_2_ 50	60.4a	2.33a	2.49a	119.5a
SiO_2_ 100	58.7ab	2.22ab	2.41ab	115.2ab

Letters in the same column indicate different groups according to Duncan's multiple range test at p < 0.05.

GP, RL, PL and SVI decreased significantly with increasing PEG-induced osmotic stress from 0 to -0.6 MPa (**Table 4**). However, nanopriming treatments significantly alleviated the effects of drought stress. ZnO at 50 mg L^-1^ showed the most significant increase under mild to moderate drought stress (-0.2 and -0.4 MPa) with significantly higher GP and SVI than the control. Additionally, SiO_2_ at 50 mg L^-1^ promoted root and plumule elongation and increased seedling vigour in general. The effects of nanopriming were generally attenuated under severe drought stress (-0.6 MPa), but ZnO at 50 had increased RL and SVI compared to the control group. The 100 mg L^-1^ treatments did not provide any additional benefit and often reduced vigour compared with the 50 mg L^-1^ treatments. The results indicate that low concentrations of NPs are more effective and the advantages of nanopriming are more significant in mild to severe drought stress. Salinity stress (0–12 dS m^-1^) significantly reduced germination and seedling traits, with the greatest inhibition observed at 12 dS m^-1^ (**Table 5**). At 4 and 8 dS m^-1^ (moderate salinity), ZnO at 50 mg L^-1^ notably increased GP, and SVI compared to the control. SiO_2_ at 50 mg L^-1^ enhanced root elongation and overall seedling vigor and seedlings primed with 50 mg L^-1^ doses maintained more robust growth despite increasing salinity. At 12 dS m^-1^ (severe salinity) all treatments showed reduced performance; however, ZnO at 50 mg L^-1^ retained a clear advantage in GP and SVI. NP treatments at 100 mg L^-1^ were not better than lower doses and sometimes showed minor toxicity, similar to drought treatments. In general, nanopriming significantly enhanced salinity tolerance, particularly under moderate stress, and ZnO and SiO_2_ exerted similar but dose-dependent beneficial effects. Both interaction terms were statistically significant indicating the nanopriming effect was dependent on the drought or salinity severity. ZnO at 50 mg L^-1^ and SiO_2_ at 50 mg L^-1^ significantly increased root length (RL) and seedling vigour index (SVI) under regulated and moderate drought stress than non-primed seeds. ZnO at 50 mg L^-1^ showed the best vigour under mild drought stress (-0.4 MPa) but SiO_2_ at 50 mg L^-1^ was more effective in promoting root growth under different drought intensities. Treatments with 100 mg L^-1^ showed lower efficacy, and there is room for improvement in stress (**Table 6**, **Figure 1**). The maximum germination percentage (GP) and seedling vigour index (SVI) was obtained in ZnO at 50 mg L^-1^ at salinity 4 and 8 dS m^-1^. SiO_2_ at 50 mg L^-1^ significantly improved root and plumule elongation and consequently higher seedling vigour index (SVI) values. At severe salinity levels (12 dS m^-1^), the benefits of nanopriming were significantly reduced; nevertheless, ZnO at 50 mg L^-1^ remained the most effective treatment among all other treatments. These interactions indicate that the effectiveness of nanopriming depends on both dosage and stress intensity, with 50 mg L^-^¹ being optimal for mitigating early-stage drought and salinity stress in *M. officinalis*. Drought and salinity stresses resulted in significant reductions in germination and seedling development in all tests. Nanopriming partially restored or improved germination traits under stress settings. The lower NP dosage (50 mg L^-1^) regularly surpassed the 100 mg L^-1^ dosage. In both stress trials, ZnO at 50 mg L^-1^ consistently yielded higher mean values for various germination and vigor indices; nevertheless, these patterns should be regarded as suggestive rather than as proof of statistically significant superiority over SiO_2_ treatments. (**Table 7**, **Figure 2**). Volatile profiling of the non-stressed control and eight selected nanopriming-stress treatments identified the same 11 compounds across all analyzed samples (**Table 8**). The selected treatments comprised ZnO at 50 mg L^-1^ under salinity (4 and 8 dS m^-1^) and drought (-0.2 and -0.4 MPa), and SiO_2_ at 50 mg L^-1^ under the corresponding salinity and drought levels. The identified compounds accounted for 90.02% of the total FID peak area in the control and 97.77-99.97% in the selected nanopriming-stress samples. Geranial and neral were the predominant constituents across all analyzed samples, while alloaromadendrene, geranyl acetate, 6-methyl-5-hepten-2-one, and β-caryophyllene were also among the major detected constituents (**Figure 3**). Because each selected treatment was analyzed once by GC/GC-MS (n=1), differences in relative abundance are presented descriptively and should not be interpreted as statistically significant treatment effects.

**Table 4:** Drought level mean values.

Drought level	Germination (%)	Plumule	Radicle	SVI
0.0 MPa	83.2a	3.42a	3.65a	181.4a
-0.2 MPa	69.1b	2.88b	3.11b	148.7b
-0.4 MPa	48.0c	1.76c	2.09c	96.3c
-0.6 MPa	28.4d	1.15d	1.42d	52.1d

Letters in the same column indicate different groups according to Duncan's multiple range test at p < 0.05.

**Table 5:** Salinity level mean values.

Salinity level	Germination (%)	Plumule	Radicle	SVI
0 dS/m	74.5a	2.96a	3.21a	166.1a
4 dS/m	61.2b	2.44b	2.59b	128.3b
8 dS/m	43.8c	1.76c	1.98c	92.2c
12 dS/m	33.4d	1.32d	1.61d	66.4d

Letters in the same column indicate different groups according to Duncan's multiple range test at p < 0.05.

**Table 6:** NP × Drought interaction on germination.

NP	0.0 MPa	-0.2 MPa	-0.4 MPa	-0.6 MPa
Control	79c	66f	42i	22l
ZnO 50	88a	76d	56g	35j
ZnO 100	84b	70e	50h	30k
SiO_2_ 50	86ab	74de	55g	33jk
SiO_2_ 100	82bc	71e	47h	27kl

Letters in the same column indicate different groups according to Duncan's multiple range test at p < 0.05.

**Figure 1:**
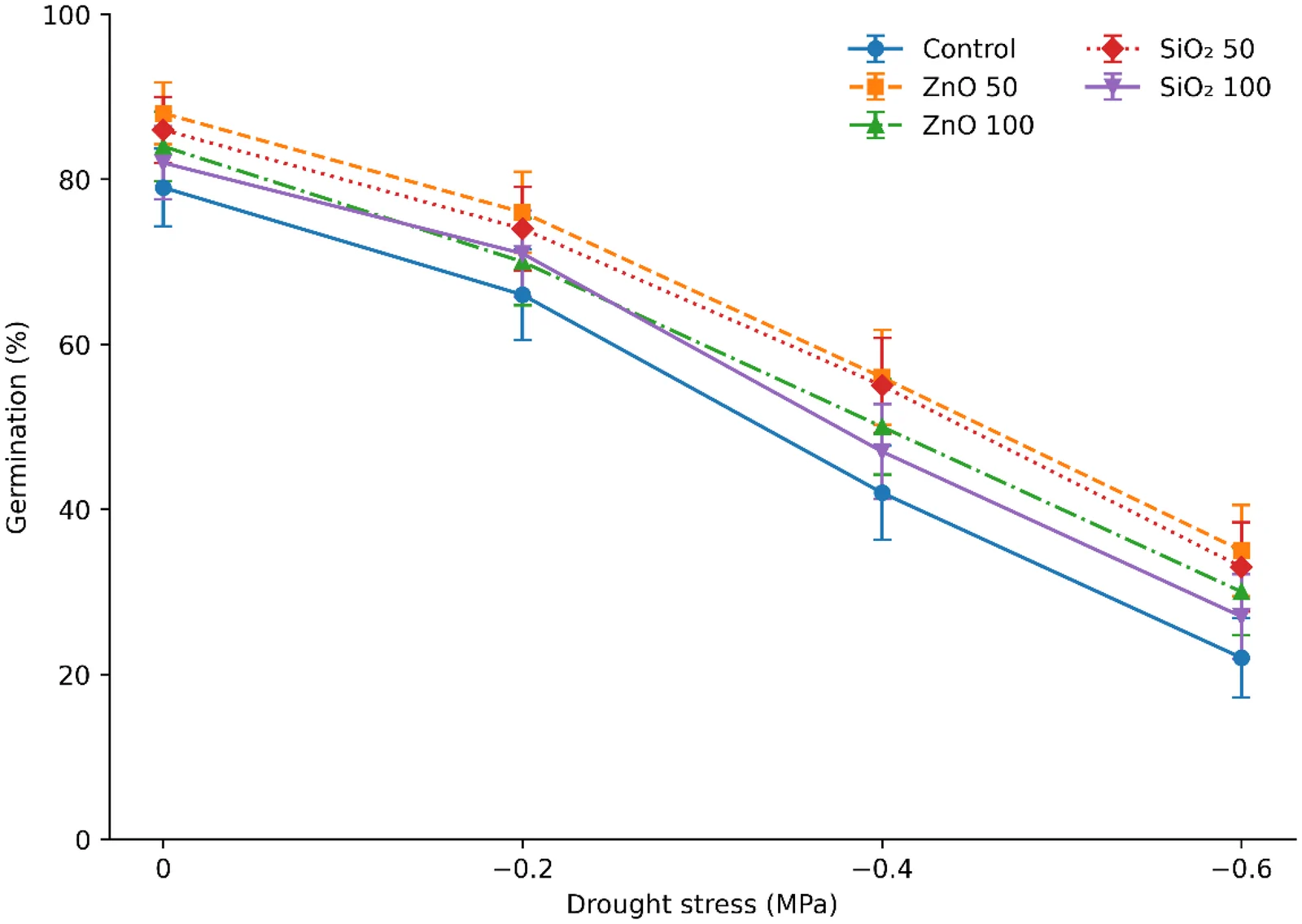
Interaction between nanoparticle treatments and drought stress on seed germination (%).

**Table 7:** NP × Salinity interaction on germination.

NP	0 dS/m	4 dS/m	8 dS/m	12 dS/m
Control	68e	55i	36l	28n
ZnO 50	82a	69e	52j	45k
ZnO 100	78b	63h	45k	36l
SiO_2_ 50	80ab	67f	49jk	42m
SiO_2_ 100	74c	59g	43l	35l

Letters in the same column indicate different groups according to Duncan's multiple range test at p < 0.05.

**Figure 2:**
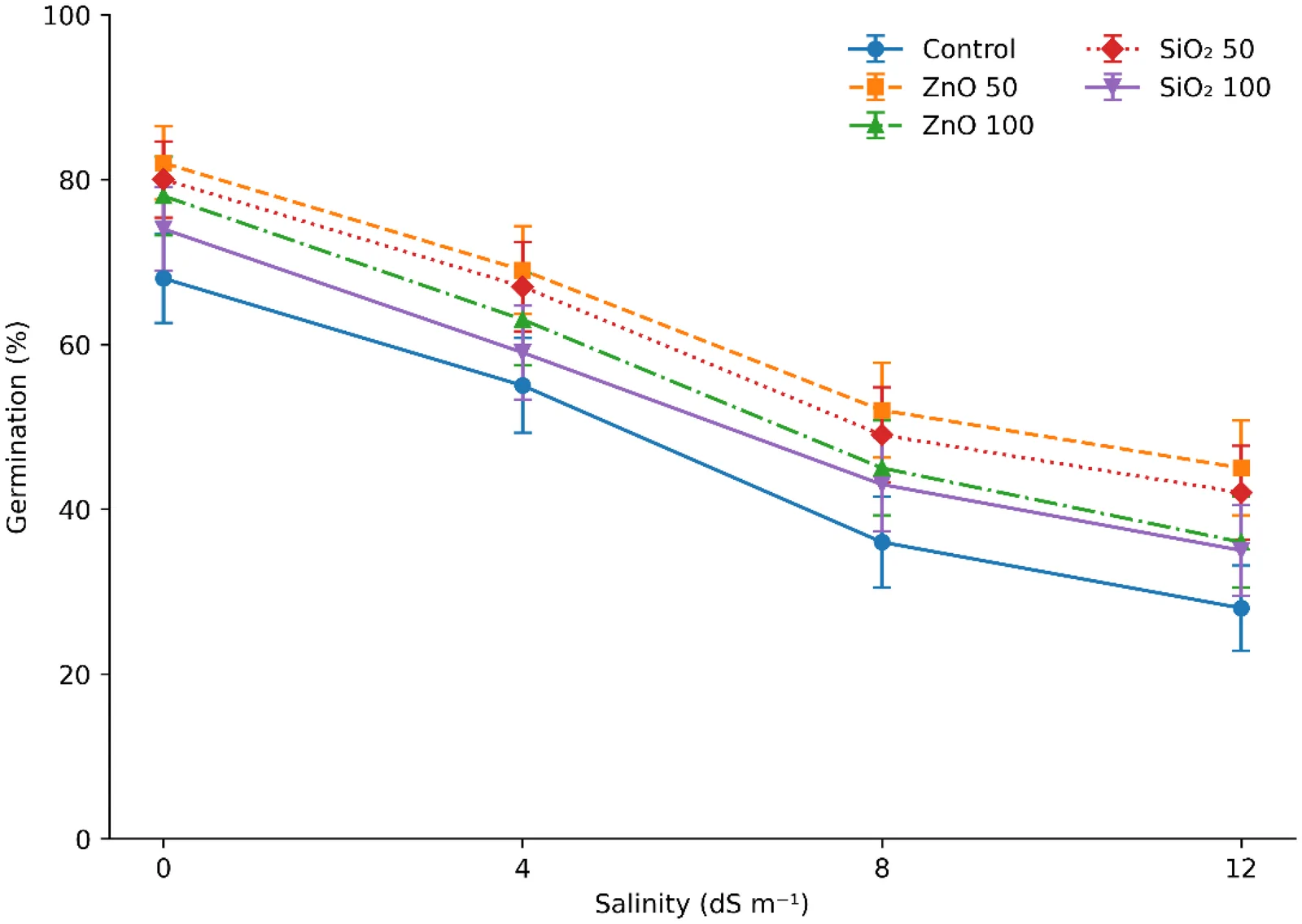
Interaction between nanoparticle treatments and salinity stress on seed germination (%).

**Table 8:** Descriptive volatile composition (%) of *Melissa officinalis* seedlings under selected nanopriming, drought, and salinity treatments.

Compounds	RRI	Control	4 dS m^-1^ + ZnO at 50 mg L^-^¹ (%)	8 dS m^-^¹ + ZnO at 50 mg L^-^¹ (%)	-0.2 MPa+ ZnO at 50 mg L^-^¹ (%)	-0.4 MPa+ ZnO at 50 mg L^-^¹ (%)	4 dS m^-1^ + SiO_2_ at 50 mg L^-^¹ (%)	8 dS m^-^¹ + SiO_2_ at 50 mg L^-^¹ (%)	-0.2 MPa+ SiO_2_ at 50 mg L^-^¹ (%)	-0.4 MPa+ SiO_2_ at 50 mg L^-^¹ (%)
*6-Methyl-5-hepten-2-one*	1344	3.4	3.7	3.8	3.7	3.7	3.6	3.7	3.8	3.7
*Linalool*	1548	0.9	1.4	1.4	1.5	1.3	1.3	1.4	1.4	1.5
*β-Caryophyllene*	1610	2.5	2.9	3.2	3.1	3.0	3.1	2.8	3.3	3.2
*Alloaromadendrene*	1659	2.4	6.6	6.5	6.3	6.5	6.7	6.6	6.8	6.6
*Neral*	1696	26.9	28.3	27.4	28.2	27.9	27.8	27.1	28.1	28.2
*(E)-Methyl geranate*	1700	1.2	1.5	1.4	1.6	1.4	1.6	1.5	1.5	1.4
*Geranial*	1738	42.5	43.9	44.1	44.3	44.2	44.2	44.1	44.8	44.2
*Geranyl acetate*	1761	7.8	7.9	7.1	7.4	7.2	7.8	7.7	7.1	7.7
*Geraniol*	1858	1.3	1.4	1.5	1.6	1.5	1.5	1.5	1.4	1.5
Ledene oxide	1903	0.91	1.27	1.31	1.39	1.44	1.41	1.37	1.49	1.51
Hexahydrofarnesyl	1923	0.21	0.23	0.27	0.25	0.29	0.24	0.26	0.28	0.29
Total (%)	–	90.02	99.10	97.98	99.34	98.43	99.25	97.77	99.97	99.80

Values represent relative peak-area percentages determined by FID from a single GC/GC-MS analysis per selected treatment (n = 1). Therefore, SD and SE values were not calculated, and the data are presented descriptively without inferential statistical comparisons. RRI, relative retention index calculated against *n-*alkanes.

**Figure 3:**
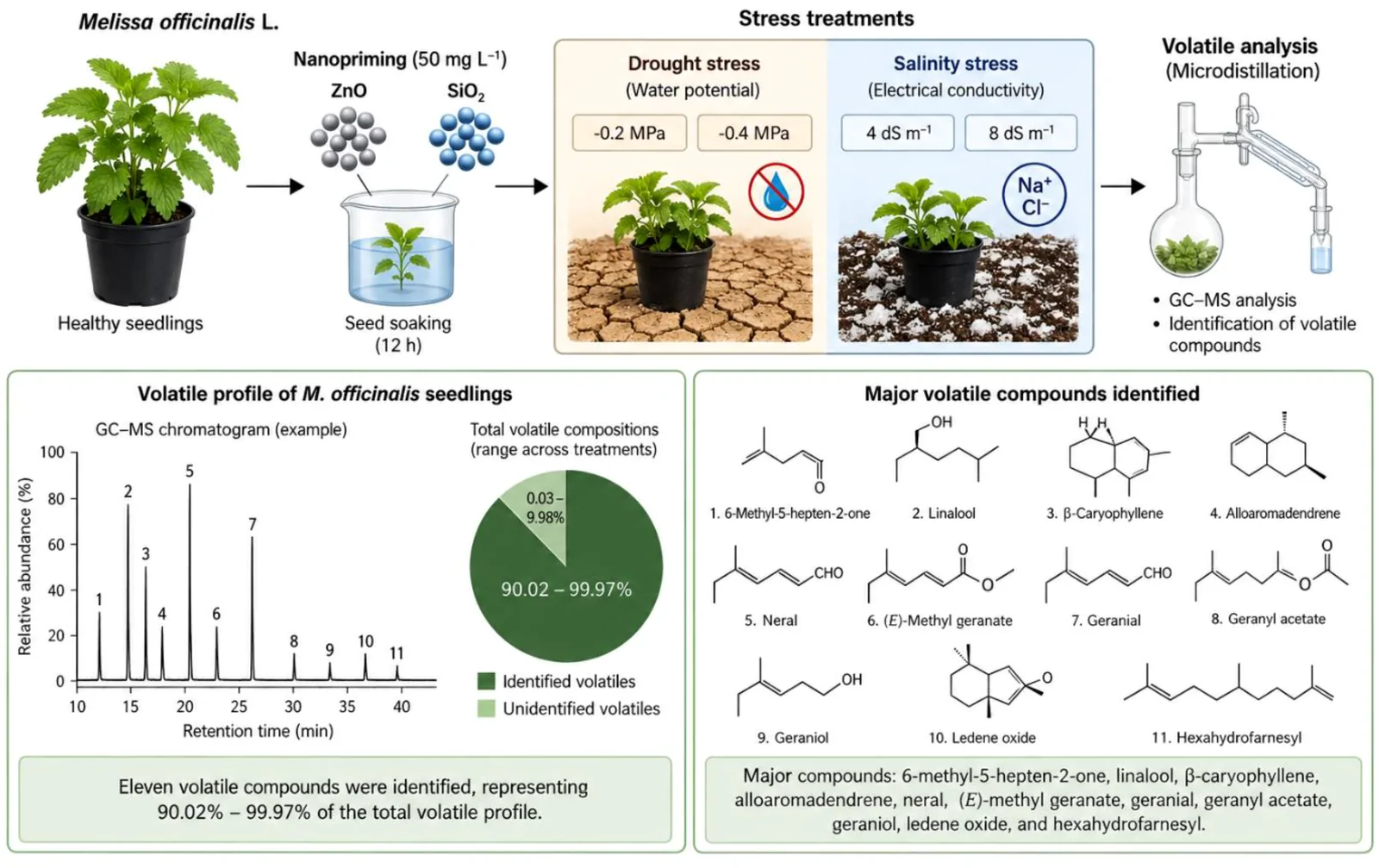
Major volatile compounds of *M. officinalis*.

## 4 Discussion

### 4.1 Effects of nanopriming on germination and seedling performance

It should be emphasized that drought and salinity were investigated in two independent factorial experiments rather than as combined stress treatments. One experiment evaluated the interaction between nanoparticle priming and PEG-induced drought stress (NP × D), whereas the other evaluated the interaction between nanoparticle priming and NaCl-induced salinity stress (NP × S). Therefore, the responses observed under drought and salinity represent independent stress-specific effects, and the present findings should not be interpreted as responses to simultaneous drought-salinity stress. The findings are consistent with the earlier research conducted by Jafari et al. (2022). By boosting free radical scavenging activity, the study showed that seed priming *Origanum majorana* with 20 and 40 mg L^-1^ of bulk and nanoparticle TiO_2_ is an efficient way to improve marjoram seedling growth characteristics under saline environments. According to Abbasi Khalaki et al. (2021), in most medicinal and forage plants, nano-priming enhanced seed germination, seedling growth and development, vigour, rate of seedling emergence, and future performance. According to Hasanaklou et al. (2023), stevia seedlings that were nano-primed exhibited noticeably greater germination indices. The germination rate in nano-primed seedlings rose by 106% to 68% (relative increase). Additionally, catalase (CAT) activity (36.15 U mg^-1^ protein) and peroxidase (POX) activity (approximately 0.057 U.mg^-1^ protein) were increased by seed priming with nSiO_2_ (I) at 10 ppm concentration. However, take note that oxidative damage was generated by nano-priming at the maximal concentration, as evidenced by an increase in H_2_O_2_ concentration. Compared to nSiO_2_ (I) and nSiO_2_ (II), bSiO_2_ had less of an impact on the enhancement of germination, seedling growth, antioxidant activities, and biochemical features. The concentration of essential oils was highest in *O. onites*. As the concentration of NaCl rose, so did the concentration (Stefanakis et al., 2024). Overall, *O. dictamnus* and *O. onites* maintained greater growth and essential oil quality indicators, indicating that they were more resilient to these stressors than *M. spicata*. Increasing salinity reduced the number of branches per plant, blooms per plant, peduncle length, and head diameter, according to Razmjoo et al. (2008). Similarly, the weight and relative volatile composition of both fresh and dry flowers were significantly decreased by adding salt. Drought circumstances resulted in significant reductions in plant height, number of branches and flowers, peduncle length, head diameter, fresh and dry flower weight, and relative volatile content. Under non-salinity stress (control) and two-day interval watering, the highest values of floral dry weight and relative volatile composition were noted.

### 4.2 Effects on volatile compound profiles

GC-MS examination of *Pancratium maritimum* L. following various nanopriming treatments revealed a shift in alkaloid profile, including treatment-dependent variations in pancratistatin and lycorine, as well as changed antioxidant content (Allam et al., 2024). Ten and eleven volatile compounds were found in the essential oil of rosemary plants that were watered with tap water (TW) and salt water (SW), respectively, according to GC/MS analysis (Sarmoum et al., 2019). According to Khorasaninejad et al. (2016), stem length, shoot wet weight, root wet weight, root dry weight, and the length of root and leaf essential oil content all dropped as salt increased. The most essential oil is present at the second salinity treatment level. Control plants with 80% field capacity had the highest concentrations of flavonoids, phenolics, and antioxidant activity (Hosseini and Heidari, 2025). As the amount of water declined, so did the total protein and chlorophyll content. Pino et al. (1999) used GC/MS to examine the essential oil of dried M. officinalis grown in Cuba, which was primarily composed of geranial (41.0%) and neral (29.9%). Citronellal, neral, geranial, and β-caryophyllene were the main constituents of the essential oil, according to Shams Ardekani et al. (2005).

## 5 Conclusions

This study demonstrates the concentration and stress-dependent efficacy of ZnO and SiO_2_ nanopriming in *Melissa officinalis*. Factorial ANOVA revealed significant effects of nanoparticle treatment and stress level on germination percentage (GP), radicle length (RL), plumule length (PL), and seedling vigour index (SVI), as well as significant NP×drought and NP×salinity interactions. This suggests that the magnitude of the nanopriming response was dependent on stress severity. Nanoparticle treatments with 50 mg L^-1^ ZnO showed the highest overall mean GP (62.1%) and SVI (124.3) compared to the non-primed control (52.4% and 96.4, respectively). However, ZnO at 50 mg L^-1^ and SiO_2_ at 50 mg L^-1^ were in the same statistical group for these features, therefore their overall effects were not statistically different. In addition, the interaction data revealed the realistic scale of the response. Under drought stress (-0.4 MPa), ZnO at 50 mg L^-1^ boosted germination from 42% to 56%, and under salt stress (8 dS m^-1^), it rose from 36% to 52%. At the greatest stress conditions, ZnO at 50 mg L^-1^ sustained germination at 35% under -0.6 MPa drought and 45% under 12 dS m^-1^ salinity, compared to 22% and 28% in the corresponding controls. The results show that 50 mg L^-1^ is the most beneficial concentration in the studied range. However, the size of the benefit is stress intensity-dependent due to the substantial NP-stress correlations. Also the same 11 volatile compounds were detected in both the non-stressed control and the eight nanopriming-stress treatments at 50 mg L^-1^. Geranial and neral were the main components, with relative percentages of 43.9-44.8% and 27.1-28.3% respectively, vs. 42.5% and 26.9% in the control. Alloaromadendrene showed the greatest descriptive change, increasing from 2.4% in the control to 6.3-6.8% throughout the nanopriming-stress treatments. The chemical data thus show changes in the relative volatile profile rather than the production of new significant molecules. These compositional changes are descriptive and cannot be regarded as statistically significant treatment effects because GC/GC-MS analysis was only performed once for each chosen treatment (n=1). From an agronomic standpoint, the findings indicate low-dose nanopriming, particularly ZnO at 50 mg L^-1^, as a potential method for improving early establishment of *M. officinalis* subjected to drought or salinity. The maintenance of germination and seedling vigour, the apparent constancy of geranial and neral as major volatiles, and the described change in alloaromadendrene may all be relevant to the production of lemon balm for medicinal, aromatic, and essential oil uses. However, these findings should not yet be interpreted as a field-level suggestion. Experiments were carried out in a controlled laboratory environment, nanoparticles were not independently characterised using techniques such as TEM/SEM, DLS, XRD, or zeta-potential analysis, volatile profiling was limited to selected 50 mg L^-1^ treatments at intermediate stress levels, stress-only unprimed controls corresponding to each selected drought and salinity level were excluded from the volatile analysis, and chemical analyses were unreplicated. Future research should validate the optimal nanoparticle concentration under greenhouse and field conditions, conduct replicated volatile analyses with appropriate stress-only controls, independently characterise nanoparticle size, morphology, and surface properties, and investigate physiological and molecular responses such as gene expression associated with stress tolerance and volatile biosynthesis. Drought and salinity were evaluated independently in the current study, so future experiments will be interesting to see if similar nanopriming responses are retained when both conditions are present at the same time.

## Data Availability

The original contributions presented in the study are included in the article/supplementary material. Further inquiries can be directed to the corresponding author.
